# THE RESIDENTIAL CARE TRANSITION MODULE: SUPPORTING DEMENTIA CAREGIVERS FOLLOWING INSTITUTIONALIZATION

**DOI:** 10.1093/geroni/igae098.1254

**Published:** 2024-12-31

**Authors:** Joseph Gaugler, Marie Boltz

**Affiliations:** Chair; Discussant

## Abstract

Families often continue to engage in various care activities following relatives’ entry to residential long-term (RLTC) care settings such as nursing homes. Dementia caregivers’ emotional and psychological adaptation to this transition is variable. We conducted rigorous, mixed methods randomized controlled evaluation to ascertain the efficacy of a remotely delivered, psychosocial/psychoeducational intervention when helping caregivers navigate the placement transition of relatives living with Alzheimer’s disease and Alzheimer’s disease-related dementias. The Residential Care Transition Module (RCTM) provided six formal sessions of consultation (one-to-one and family sessions) over a 4-month period to family caregivers who have admitted a relative living with dementia to an RLTC setting as well as ongoing, “ad hoc” consultation over an additional eight months. This symposium presents the quantitative, qualitative, and mixed methods findings of this dementia care intervention.

